# New Compounds from the Roots of Corsican *Calicotome Villosa* (Poir.) Link.: Two Pterocarpans and a Dihydrobenzofuran

**DOI:** 10.3390/molecules25153467

**Published:** 2020-07-30

**Authors:** Doreen Stacy Palu, Mathieu Paoli, Hervé Casabianca, Joseph Casanova, Ange Bighelli

**Affiliations:** 1 Department of Chemistry, Equipe Chimie-Biomasse, Université de Corse-CNRS, UMR 6134 SPE, Route des Sanguinaires, F- 20000 Ajaccio, France; palu_d@univ-corse.fr (D.S.P.); joseph.casanova@wanadoo.fr (J.C.); bighelli_a@univ-corse.fr (A.B.); 2 Institut des Sciences Analytiques, Université de Lyon, CNRS, Université Claude Bernard Lyon 1, UMR 5280, 5 rue de la Doua, F-69100 Villeurbanne, France; herve.casabianca@isa-lyon.fr

**Keywords:** *Calicotome villosa*, root extract, pterocarpan, dihydrobenzofuran, 1D and 2D NMR

## Abstract

Three new compounds, a dihydrobenzofuran (coumaran) derivative (compound **1**) and two pterocarpans (compounds **2** and **3**) were isolated from a root extract of *Calicotome villosa* growing wild in Corsica. Their structures were elucidated using 1D and 2D NMR spectroscopy and MS/MS as 2-(1-methylethenyl)-5-hydroxy-6-carbomethoxy-2,3-dihydro-benzofuran, 4,9-dihydroxy-3-methoxy-2-dimethylallylpterocarpan, and 4,9-dihydroxy-3′,3′-dimethyl-2,3-pyranopterocarpan.

## 1. Introduction

*Calicotome villosa* (Poir.) Link. (Fabaceae) is a shrub that can reach 2 m in high, with grey-tomentose stems and sharp terminations, villous pods, trifoliate and oval leaves, and yellow and grouped flowers during the spring season [1,2]. It is very common in the Mediterranean area and particularly in Corsica Island, where it grows near the sea, while the subspecies *C. villosa* subsp. *intermedia* is distributed especially in the North of Africa and Spain [3]. Solvent extracts, essential oil, and isolated compounds from *C. villosa* showed diverse biological activities including antioxydant, antimicrobial, anti-inflammatory, antidiabetic, hypotensive, diuretic, and vasodilatator [4,5,6,7]. Phytochemicals that belong to various families have been isolated and identified in *C. villosa* extracts from different parts of the plant, including alkaloids, flavonoids, steroids, anthraquinones, and phenol derivatives [2,3,4,7,8,9,10,11,12]. In addition, falcarinol (fatty alcohol), some oxygenated mono- and sesquiterpenes, furan derivatives, paraffins, and fatty acids have been identified in the essential oil from aerial parts of *C. villosa* [2,4,5]. Concerning root extracts, only one paper reported on the screening of total alkaloid, saponin, and flavonoid contents [13].

Identification of individual components of *C. villosa* root extracts has not yet been reported. In continuation of our investigations on the constituents of Corsican aromatic and medicinal plants, we report in this paper on the isolation and structural elucidation of three new compounds contained in *C. villosa* roots.

## 2. Results and Discussion

Air-died roots of *C. villosa* were extracted with methanol (soxhlet apparatus), yielding a methanolic extract (4.7%) after evaporation of the solvent. This extract was partitioned by column chromatography (CC) with hexane (F1), dichloromethane (F2), ethyl acetate (F3), and methanol (F4). Repetitive column chromatography was carried out on the dichloromethane fraction (F2) and ethyl acetate fraction (F3):

- Compound **1**, the major component of fraction F2, was isolated in pure form by repetitive CC and its structure elucidated using a combination of spectroscopic techniques including 2D NMR “INADEQUATE” (Incredible Natural Abundance DoublE QUAntum Transfer Experiment) sequence.
- As observed on their ^13^C NMR spectra, the sub-fractions F3.B.3 and F3.B.7 (see experimental) contained only the unidentified compounds **2** and **3** with the relative ratios 3:1 and 1:4, respectively, according to the relative intensities of signals. Although we did not succeed in isolating **2** and **3** in a pure form, the full set of 1D and 2D NMR experiments was conducted on the sub-fractions and compounds **2** and **3** were unambiguously identified.

### 2.1. Determination of 2-(1-methylethenyl)-5-hydroxy-6-carbomethoxy-2,3-dihydrobenzofuran (***1***)

The ^13^C NMR spectrum of sub-fraction F2.5 from dichloromethane fraction (F2) displayed 13 signals that were differentiated by DEPT (Distortionless Enhancement by Polarization Transfer) experiment as 6 C, 3 CH, 2 CH_2_, and 2 CH_3_ (Figures S1 and S2). The exact mass ([M + Na]^+^, C_13_H_14_O_4_Na^+^, experimental mass = 257.0783, theoretical mass = 257.0789) calculated using MS/MS suggested the molecular formula C_13_H_14_O_4_ (monoisotopic neutral mass = 234.0892), in agreement with data from the DEPT spectrum. Eight signals between 107.40 ppm and 156.86 ppm belonged to carbons of four double bonds, of which one included a methylidene group (=CH_2_; 112.23 ppm). The presence of signals at δC = 170.63 ppm (quaternary carbon) and at δC = 52.21 ppm, δH = 3.91 ppm (3H), indicated the occurrence of a carbomethoxy group. According to the seven degrees of unsaturation, compound **1** contained two rings. The two singlets at δH = 6.80 ppm and δH = 7.16 ppm suggested a tetra-substituted phenyl moiety with hydrogens in para position (Figure S3). It was assumed that the second ring belonged to a dihydrofuran sub-structure (δC = 85.52 ppm, δH = 5.13 ppm). Lastly, the hydrogen atom at δH = 10.55 ppm (singlet) belonged to a hydroxyl group, probably a phenoxy group. Interpretation of 2D NMR spectra, *i.e.*, HSQC (Heteronuclear Single Quantum Coherence), HMBC (Heteronuclear Multiple Bond Correlation), COSY (COrrelation SpectroscopY), and NOESY (Nuclear Overhauser Effect SpectroscopY), allowed for the positioning of all substituents on the dihydrobenzofuran skeleton, leading to the structure of compound **1** (Table 1, Figures S4–S8). For instance, correlations between the proton at 10.55 ppm (OH) and carbons C4 (CH; 113.79 ppm), C5 (C; 156.86 ppm), and C6 (C; 110.48 ppm) indicated that the hydroxyl group is connected to C5. Consequently, quaternary carbon C6 bears the carbomethoxy group. Analysis of the INADEQUATE spectrum confirmed the structure. Some carbon–carbon correlations were particularly informative (Figures S9 and S10). For instance, correlations between the methine at δC = 85.52 ppm (C2) and the quaternary carbon at δC = 143.67 ppm (C10) showed that the isopropylene (methylethenyl) group is linked to the dihydrobenzofuran skeleton at C2 (Figure S9). The structure of compound **1** was elucidated as 2-(1-methylethenyl)-5-hydroxy-6-carbomethoxy-2,3-dihydrobenzofuran (Table 1, Figures S1–S11). Compound **1** is an isomer of methyl tubaiate; however, the two compounds are easily differentiated by their ^1^H NMR data. Indeed, aromatic protons of methyl tubaiate appear as two doublets at 7.69 ppm and 6.37 ppm (J = 9 Hz) [14] while the signals of aromatic protons of compound **1** are two singlets at 6.80 ppm and 7.16 ppm.

### 2.2. Determination of 4,9-dihydroxy-3-methoxy-2-dimethylallylpterocarpan (***2***)

Although compound **2** could not be obtained in pure form, its degree of purity allowed for its structural elucidation. Indeed, the ^13^C NMR spectrum of sub-fraction F3.B.3 from EtOAc fraction (F3) displayed a series of 21 signals with strong intensities that belonged to compound **2**. The main signals were accompanied by a series of much smaller signals, which belonged to compound **3**, as demonstrated in the next paragraph.

Exact mass obtained from the MS/MS analyses ([M + Na]^+^, C_21_H_22_O_5_Na^+^, experimental mass = 377.1347, theoretical mass = 377.1359) for compound **2** led to the molecular formula C_21_H_22_O_5_ (monoisotopic neutral mass = 354.1467) in agreement with ^13^C NMR and DEPT spectra (Table 2). The formula of this compound displayed 11 degrees of unsaturation.

According to ^13^C NMR chemical shifts, the molecule contained 14 sp^2^ carbons (seven double bonds) and therefore four cycles.

The ^1^H NMR spectrum displayed the sub-structure -O-CH_2_-CH-CH-O- (4.32 ppm, dd; 3.62 ppm, t; 3.57 ppm, m; 5.50 ppm, d) included in a “pyrano-furano” framework (Table 2). HSQC spectrum evidenced that the chemical shifts of the corresponding carbons were 67.04 ppm, 39.70 ppm, and 78.44 ppm. Various correlations in the HMBC spectrum confirmed the sub-structure: (i) proton H6 with C4a, C6a, C6b, and C11a; (ii) proton H6a with C6, C6b, C7, and C10a; and (iii) proton H11a with C1, C4a, C6, C6a, and C11b. Other correlations in the HMBC spectrum suggested the occurrence of two phenyl moieties and an isolated double bond.

Chemical shifts, multiplicities of signals, and coupling constant values are characteristic of the pterocarpan skeleton [15]. In the ^13^C NMR spectrum, the occurrence of 12 out of 14 signals belonging to sp^2^ carbons confirmed the pterocarpan skeleton that includes two phenyl moieties (A and D cycles; Table 2). Moreover, the value of the ^3^J coupling constant between H6a and H11a (6.6 Hz) confirmed the cis junction of the two aliphatic cycles, characteristic of a pterocarpan skeleton [16], in agreement with the correlation plots observed in the COSY and NOESY spectra (Table 2).

Lastly, the remaining signals are characteristic of the γ, γ’-dimethylallyl substructure, corroborated by appropriate correlations in the HMBC spectrum. In that spectrum, correlations between H1′ and C1, C2, and C3 allowed the positioning of the γ, γ’-dimethylallyl group on carbon 2 of cycle A of the pterocarpan.

^1^H and ^13^C NMR spectra evidenced also a methoxy group (-O-CH_3_) as well as two “phenol” functions. The positioning of the methoxy group and the two hydroxyl functions were confirmed by correlations observed in the HMBC spectrum (Table 2, Figure S12). Indeed, HMBC correlations between the proton at 4.95 ppm (singlet) with aromatic carbons C8, C9, and C10 on the one hand, and the proton at 5.47 ppm (singlet) with aromatic carbons C3, C4, and C4a on the other hand evidenced two hydroxyl groups linked at C9 and C4, respectively. In addition, the correlation between the signal at 3.86 ppm (3H), belonging to the methoxy group, with the quaternary carbon at 145.52 ppm indicated the positioning of this group linked at the C3 position of the ring.

Thus, compound **2** is a new pterocarpan, named 4,9-dihydroxy-3-methoxy-2-dimethylallylpterocarpan, whose full NMR data are reported in the Table 2 and Figures S12–S20 (^1^H and ^13^C NMR spectra, HSQC, HMBC, COSY, and NOESY). Isoprenyl pterocarpans have been reported as secondary metabolites from various plants belonging to the Fabaceae family, for instance *Pueraria mirifica* [17,18], as well as pterocarpans containing the dimethylbenzopyrane substructure in *Lespedeza floribunda* [19]. 2D NMR was efficient to locate the various substituents on the pterocarpan skeleton.

### 2.3. Determination of 4,9-dihydroxy-3′,3′-dimethyl-2,3-pyranopterocarpan (***3***)

Compound **3** could not be obtained in pure form, but its degree of purity allowed for its structural elucidation. Indeed, in the sub-fraction F3.B.7 compound **3** was accompanied by compound **2**, previously identified. The relative ratio evaluated by comparing the mean intensities of the signals of protonated carbons of every molecule in the spectrum was equal to 4:1. Therefore, the full set of 2D NMR experiments was conducted on fraction F3.B.7.

The ^13^C NMR spectrum of sub-fraction F3.B.7 from EtOAc fraction (F3) displayed a series of 20 signals with strong intensities that belonged to compound **3**. MS/MS analysis ([M + Na]^+^, C_20_H_18_O_5_Na^+^, experimental mass = 361.1050, theoretical mass = 361.1046) led to the molecular formula C_20_H_18_O_5_ (monoisotopic neutral mass = 338.1154). The formula of this compound displayed 12 degrees of unsaturation.

As previously observed for compound **2**, the ^1^H NMR spectrum of compound **3** evidenced the sub-structure -O-CH_2_-CH-CH-O- (4.34 ppm, dd; 3.63 ppm, t; 3.53 ppm, m; 5.48 ppm, d; Table 3). HSQC spectrum indicated that the chemical shifts of the corresponding carbons were 66.92 ppm, 39.53 ppm, and 78.57 ppm. Various correlations in the HMBC spectrum confirmed the sub-structure: (i) proton H6 with C4a, C6a, C6b, and C11a; (ii) proton H6a with C6, C6b, C7, and C10a; and (iii) proton H11a with C1, C4a, C6, C6a, and C11b.

Chemical shifts, multiplicities of signals, and coupling constant values, as well as the number of sp^2^ carbons belonging to two phenyl moieties, are characteristic of the pterocarpan skeleton [14] (Table 3). Once again, the value of the ^3^J coupling constant between H6a and H11a (6.5 Hz for compound **3**) confirmed the cis junction of the two aliphatic cycles [16].

Concerning degrees of unsaturation, eight of these belonged to two phenyl moieties and two others are due to the B and C oxygenated cycles of the pterocarpan skeleton. According to the chemical shift values of signals, the two last degrees of unsaturation corresponded to an isolated double bond on the one hand, and a fifth cycle on the other hand.

We have already seen that the molecule contained the -O-CH_2_-CH-CH-O- sub-structure and two phenyl rings characteristics of the pterocarpan skeleton. According to carbon chemical shift values, compound **3** possesses an identical D ring to that of compound **2** and other pterocarpans such as desmocarpin, erysubin C, or edudiol [21] (Table 3). Therefore, the fifth cycle is linked to the aromatic A ring and includes an oxygen atom linked to a quaternary carbon at δC = 77.31 ppm and a double bond with two coupled hydrogens at δ = 5.57 ppm and δ = 6.31 ppm (^3^J = 9.8 Hz, COSY spectrum), describing a pyrane sub-structure (or benzopyrane if cycle A is included in the sub-structure) (Figure S21). According to the HMBC correlations, the proton at 6.31 ppm (doublet, linked to the carbon at 121.79 ppm, C1′) correlates with the carbons at 118.41 ppm (methine), 116.12 ppm, and 140.30 ppm (quaternary carbons). These correlations indicated that the pyrano ring is connected with the A ring by carbons C2 and C3. Compound **3** is also a new pterocarpan, named 4,9-dihydroxy-3′,3′-dimethyl-2,3-pyranopterocarpan (full 1D and 2D NMR data reported in the Table 3, Figures S21–S29). This compound may be seen as 4-hydroxyneorautenol. The biosynthetic pathway up to compounds **2** and **3** starting from phenylalanine may be hypothesized, similar to that mentioned by Goel et al. [22].

## 3. Materials and Methods

### 3.1. Plant Material

Roots of *C. villosa* were collected in September 2018 in Ajaccio, Corsica, France (41°54′45.1′′ N; 8°39′9.8′′ E), in collaboration with the Conservatoire Botanique de Corse. Our samples of *C. villosa* were identified as similar to the voucher specimen number 12057/1 (Jeanmonod D. and Roguet D.) deposited in the Conservatoire et Jardin Botanique de Genève.

### 3.2. Extraction and Isolation

Air-dried roots of *C. villosa* (192.6 g) were first extracted (soxhlet apparatus) with methanol (500 mL) for 24 h followed by concentration under reduced pressure to afford a dark brown extract (9.18 g). This extract was chromatographed over a silica gel column (200–500 µm; 150 g) and sequentially eluted with hexane, dichloromethane, ethyl acetate, and methanol to give four fractions (F1–F4).

Fraction F2 (330 mg), eluted with dichloromethane, was subjected to a silica gel column (35–70 µm; 10 g) with a gradient of solvents (pentane/diethyl ether, 100:0 to 0:100) to give nine fractions (F2.1–F2.9)_._ The fraction F2.5 (76 mg) eluted with pentane/diethyl ether (95:5) contained compound **1** in pure form (viscous yellow liquid).

Fraction F3 (4.19 g), eluted with ethyl acetate, was chromatographed on a silica gel column (200–500 µm; 106 g) with a gradient of solvents (dichloromethane/ethyl acetate, 100:0 to 0:100) to afford 13 fractions (F3.1–F3.13). The fractions (F3.5–F3.7) having similar ^13^C NMR spectra were combined, giving F3.A (611 mg) and chromatographed once again over a silica gel (35–70 µm; 30 g) with a gradient of solvents (dichloromethane/ethyl acetate 100:0 to 50:50) to give 13 sub-fractions (F3.A.1–F3.A.13). Sub-fractions (F3.A.8–F3.A.11) having similar ^13^C NMR spectra were combined (giving F3.B, 114 mg) and subjected to a silica gel column (35–70 µm; 7 g) with a gradient of solvents (dichloromethane/ethyl acetate, 100:0 to 90:10) to give ten other sub-fractions (F3.B.1–F3.B.10). The sub-fractions F3.B.3 (21 mg) and F3.B.7 (13 mg), eluted with dichloromethane/ethyl acetate (99.5:0.5), afforded compounds **2** and **3** in ratios 3:1 and 1:4, respectively. These fractions were used for structure elucidation of compounds **2** and **3**. It could be assumed that compounds **1**–**3**, isolated using standard procedures, are secondary metabolites of the plant and are not artifacts from the isolation process.

### 3.3. NMR Spectroscopy

All NMR spectra were recorded on a Bruker AVANCE 400 spectrometer (400.132 MHz for ^1^H; 100.623 MHz for ^13^C), equipped with a 5 mm probe, in deuterated chloroform (CDCl_3_) or deuterated dimethylsulfoxide (DMSO-*d*_6_), with all chemical shifts referred to internal tetramethylsilane (TMS) for spectra recorded in CDCl_3_ and to the signal of solvent (39.39 ppm for ^13^C and 2.50 ppm for ^1^H) for spectra recorded in DMSO-d6. ^1^H NMR spectra were recorded with the following parameters: flip angle 30°; acquisition time 2.56 s for 32 K data table with a spectral width of 6410 Hz (16 ppm); relaxation delay D_1_ = 1 s. ^13^C NMR spectra were recorded with the following parameters: flip angle 45°; acquisition time 2.66 s for 128 K data table with a spectral width of 25,000 Hz (250 ppm); relaxation delay D_1_ = 0.1 s; CPD mode decoupling; digital resolution 0.183 Hz/pt. The number of accumulated scans was 1000–3000, depending of the available amount of fraction of CC (5–50 mg in 0.5 mL of solvent: CDCl_3_ for F1, F2, F2.1–2.9, F3.1–3.9, F3.A.1–3.A.12, F3.B.1–3.B.10; DMSO-*d*_6_ for F3, F4, F3.10–3.13 and F3.A.13). DEPT spectra were recorded with the same parameters as ^13^C NMR spectra, except flip angle (135°). Standard Bruker pulse sequences were used for COSY, NOESY, INADEQUATE, HSQC, and HMBC experiments.

### 3.4. Mass Spectrometry

MS/MS analyses were obtained with Bruker Daltonics Maxis Plus, Data analysis 4.3. Direct infusion by ESI-MS and confirmation by MS/MS with preferred mass list. A solution of sodium formate and acetate 10 mM clusters was used for the calibration. The analyses were performed over a mass range of 50–1000 Daltons with scan rate of 1 Hz. The instrument resolution was estimated 21,244 (FWHM) at m/z = 415.211. Products were dissolved in methanol for direct introduction. Compound **1** m/z (%): 257.0783 ([M + Na]^+^ 100); 256.0718 (36.3); 225.0522 (27.9); 62.9816 (26.8). Compound **2** m/z (%): 377.1347 ([M+Na]^+^ 9.4); 308.0651 (29); 293.0407 (24.4); 164.9189 (30.1); 149.0592 (35.1); 105.0333 (17.6); 90.9757 (39.8); 69.0684 (8.3); 62.9804 (100); 56.0480 (49.5). Compound **3**: m/z (%): 361.1050 ([M + Na]^+^ 46.3); 313.2039 (10.8); 164.9197 (26.4); 104.9923 (6.2); 91.0541 (5.0); 62.9803 (100) (fragmentation pattern observed in the mass spectra similar to that reported by Simons et al. [23] for other pterocarpans, Figure S30).

## 4. Conclusions

Three new compounds were isolated from the roots of *C. villosa* growing wild in Corsica (France) and their structures elucidated as 2-(1-methylethenyl)-5-hydroxy-6-carbomethoxy-2,3-dihydrobenzofuran (**1**), 4,9-dihydroxy-3-methoxy-2-dimethylallylpterocarpan (**2**), and 4,9-dihydroxy-3′,3′-dimethyl-2,3-pyranopterocarpan (**3**). Various pterocarpan derivatives were identified in root extracts from Fabaceae, some of which are known as bioactive compounds. These compounds hold great potential for therapeutic treatment. Lastly, to the best of our knowledge, dihydrobenzofuran derivatives have rarely been identified in *Calicotome* species extracts.

## Figures and Tables

**Table 1:** Structure and NMR data of compound **1**.

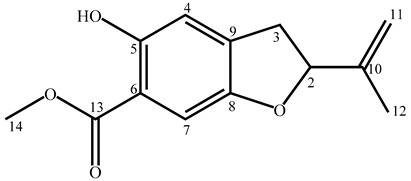							
N°C	δ^13^C (ppm)	DEPT	δ^1^H (ppm)	Multiplicity (J (Hz))	HMBC (H→C)	COSY	NOESY
2	85.52	CH	5.13	t (8.0)	11	3a; 3b	3a; 3b; 12
3	35.27	CH_2_	3.02 (a) 3.33 (b)	ddd (16.7; 8.0; 1.2) ddd (16.7; 8.0; 1.2)	2; 4; 5; 8; 9; 10	2; 3b; 4 2; 3a; 4	2; 4; 12 2; 4; 12
4	113.79	CH	6.80	br s	3; 5; 6; 7; 8; 9; 13	3a; 3b	3a; 3b; 5-OH
5	156.86	C	-	-	-	-	-
6	110.48	C	-	-	-	-	-
7	107.40	CH	7.16	s	4; 5; 6; 8; 9; 13	-	-
8	152.34	C	-	-	-	-	-
9	136.55	C	-	-	-	-	-
10	143.67	C	-	-	-	-	-
11	112.23	CH_2_	4.91(a) 5.07(b)	m m	2; 10; 12 2; 3; 10; 12	12	11b; 12 11a; 12
12	17.17	CH_3_	1.75	br s	2; 10; 11	11a; 11b	2; 3a; 3b; 11a; 11b
13	170.63	C	-	-	-	-	-
14	52.21	CH_3_	3.91	s	13	-	-
5-OH	-	-	10.55	s	4; 5; 6; 9	-	4

Notes: s = singlet, d = doublet, t = triplet, m = multiplet, br = broad; hydrogens H3a/H3b and H11a/H11b could not be differentiated; numbering according to Obara et al. [14].

**Table 2:** Structure (relative stereochemistry) and NMR data of compound **2**.

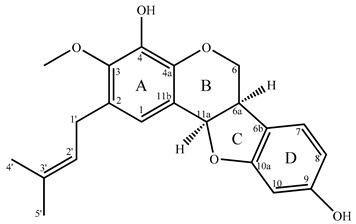							
N°C	δ^13^C (ppm)	DEPT	δ^1^H (ppm)	Multiplicity (J (Hz))	HMBC (H→C)	COSY	NOESY
1	121.00	CH	6.89	br s	1′; 3; 4; 4a; 11a	-	1′; 11a
2	128.72	C	-	-	-	-	
3	145.52	C	-	-	-	-	
4	137.72	C	-	-	-	-	
4a	142.01	C	-	-	-	-	
6	67.04	CH_2_	4.32 (x) 3.62 (y)	dd (10.9;4.8) t (10.9)	4a; 6a; 6b; 11a	6y; 6a 6x;	6y 6x
6a	39.70	CH	3.57	m	6; 6b; 7; 10a	11a; 6x	7; 11a
6b	118.99	C	-	-	-	-	
7	125.01	CH	7.08	d (8.6)	6a; 9; 10; 10a	8	6a; 8
8	107.75	CH	6.37	m	10; 10a	7	7
9	157.02	C	-	-	-	-	
10	98.48	CH	6.39	s	6b;8;9	-	9-OH
10a	160.70	C	-	-	-	-	
11a	78.44	CH	5.50	d (6.6)	1; 4a; 6; 6a; 11b	6a	1; 6a
11b	115.52	C	-	-	-	-	
1′	28.15	CH_2_	3.33	m	1; 2; 2′; 3; 3′	2′; 4′; 5′	1; 2′; -OCH_3_
2′	122.81	CH	5.29	m	4′; 5′	1′; 4′; 5′	1′
3′	132.46	C	-	-	-	-	
4′	17.85	CH_3_	1.74	br s	2′; 3′; 5′	1′; 2′	
5′	25.83	CH_3_	1.74	br s	2′; 3′; 4′	1′; 2′	
-OCH_3_	60.62	-	3.86	s	3	-	1′
4-OH	-	-	5.47	s	3; 4; 4a	-	-
9-OH	-	-	4.95	s	8; 9; 10	-	10

Notes: s = singlet, t = triplet, d = doublet, m = multiplet, br = broad; numbering according to Ingham and Tahara [20].

**Table 3:** Structure (relative stereochemistry) and NMR data of compound **3**.

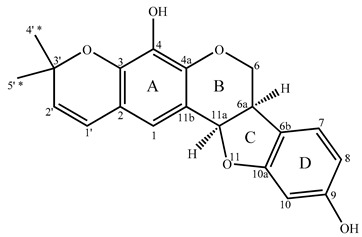							
N°C	δ^13^C (ppm)	DEPT	δ^1^H (ppm)	Multiplicity (J (Hz))	HMBC (H→C)	COSY	NOESY
1	118.41	CH	6.77	s	1′; 2; 3; 4; 4a; 11a	-	1′; 11a
2	116.12	C	-	-	-	-	
3	140.30	C	-	-	-	-	
4	133.01	C	-	-	-	-	
4a	143.73	C	-	-	-	-	
6	66.92	CH_2_	4.34 (x) 3.63 (y)	dd (10.8; 5.0) t (10.8)	4a; 6a; 6b; 11a	6y; 6a 6x	6y; 6a 6x;
6a	39.53	CH	3.53	m	6; 6b; 7; 10a; 11a	11a; 6x	6x; 11a
6b	119.00	C	-	-	-	-	-
7	125.04	CH	7.08	d (8.3)	6; 6a; 6b; 9; 10; 10a	8	8
8	107.77	CH	6.37	m	6b	7	7
9	157.07	C	-	-	-	-	
10	98.44	CH	6.38	s	6b; 8; 9; 10a	-	
10a	160.63	C	-	-	-	-	
11a	78.57	CH	5.48	d (6.5)	1; 2; 4a; 6; 6a; 11b	6a	1; 6a
11b	112.65	C	-	-	-	-	
1′	121.79	CH	6.31	d (9.8)	1; 2; 3; 3′; 4; 5′	2′	1; 2′
2′	129.39	CH	5.57	d (9.8)	2; 3; 3′; 4′	1′	1′; 4′; 5′
3′	77.31	C	-	-	-	-	-
4′ *	28.16	CH_3_	1.48	s	1′; 2′; 3; 3′; 5′		2′
5′ *	27.90	CH_3_	1.44	s	1′; 2′; 3′; 4′		2′
4-OH	-	-	5.38 ^#,$^	s	-	-	-
9-OH	-	-	5.28 ^#,$^	m	-	-	-

Notes: s = singlet, d = doublet, m = multiplet; br = broad; ^*,#^: signal could be interchanged; ^$^ = correlation plot not observed on the HMBC NMR spectrum; numbering according to Ingham and Tahara [20].

## Supplementary Materials

The following are available online. Figure S1: ^13^C NMR spectrum of fraction F2.5 (compound **1**), Figure S2: DEPT 135 NMR spectrum of fraction F2.5 (compound **1**), Figure S3: ^1^H NMR spectrum of fraction F2.5 (compound **1**), Figure S4: Structure of compound **1** with key HMBC and COSY correlations, Figure S5: HSQC NMR spectrum of fraction F2.5 (compound **1**), Figure S6: HMBC NMR spectrum of fraction F2.5 (compound **1**), Figure S7: COSY NMR spectrum of fraction F2.5 (compound **1**), Figure S8: NOESY NMR spectrum of fraction F2.5 (compound **1**), Figure S9: Structure of compound **1** with 2D NMR INADEQUATE correlations, Figure S10: INADEQUATE NMR spectrum of fraction F2.5 (compound **1**), Figure S11: MS/MS spectrum of compound **1**, Figure S12: Structure of compound **2** with key HMBC and COSY correlations, Figure S13: ^1^H NMR spectrum of sub-fraction F3.B.3 (compound **2**/compound **3** = 3:1), Figure S14: ^13^C NMR spectrum of sub-fraction F3.B.3 (compound **2**/compound **3** = 3:1), Figure S15: DEPT 135 NMR spectrum of sub-fraction F3.B.3 (compound **2**/compound **3** = 3:1), Figure S16: HSQC NMR spectrum of sub-fraction F3.B.3 (compound **2**/compound **3** = 3:1), Figure S17: HMBC NMR spectrum of sub-fraction F3.B.3 (compound **2**/compound **3** = 3:1), Figure S18: COSY NMR spectrum of sub-fraction F3.B.3 (compound **2**/compound **3** = 3:1), Figure S19: NOESY NMR spectrum of sub-fraction F3.B.3 (compound **2**/compound **3** = 3:1), Figure S20: MS/MS spectrum compound **2**, Figure S21: Structure of compound **3** with key HMBC and COSY correlations, Figure S22: ^1^H NMR spectrum of sub-fraction F3.B.7 (compound **2**/compound **3** = 1:4), Figure S23: ^13^C NMR spectrum of sub-fraction F3.B.7 (compound **2**/compound **3** = 1:4), Figure S24: DEPT 135 NMR spectrum of sub-fraction F3.B.7 (compound **2**/compound **3** = 1:4), Figure S25: HSQC NMR spectrum of sub-fraction F3.B.7 (compound **2**/compound **3** = 1:4), Figure S26: HMBC NMR spectrum of sub-fraction F3.B.7 (compound **2**/compound **3** = 1:4), Figure S27: COSY NMR spectrum of sub-fraction F3.B.7 (compound **2**/compound **3** = 1:4), Figure S28: NOESY NMR spectrum of sub-fraction F3.B.7 (compound **2**/compound **3** = 1:4), Figure S29: MS/MS spectrum compound **3**, Figure S30: Possible pathway cleavages of compounds **2** and **3**.
