# Supplementary material for: New Compounds from the Roots of Corsican Calicotome Villosa (Poir.) Link.: Two Pterocarpans and a Dihydrobenzofuran
Source: Molecules. 2020 Jul 30;25(15):3467. doi: 10.3390/molecules25153467 (PMC7435676; doi:10.3390/molecules25153467)

Figure S1. ^{13}C NMR spectrum of fraction F2.5 (compound **1**).

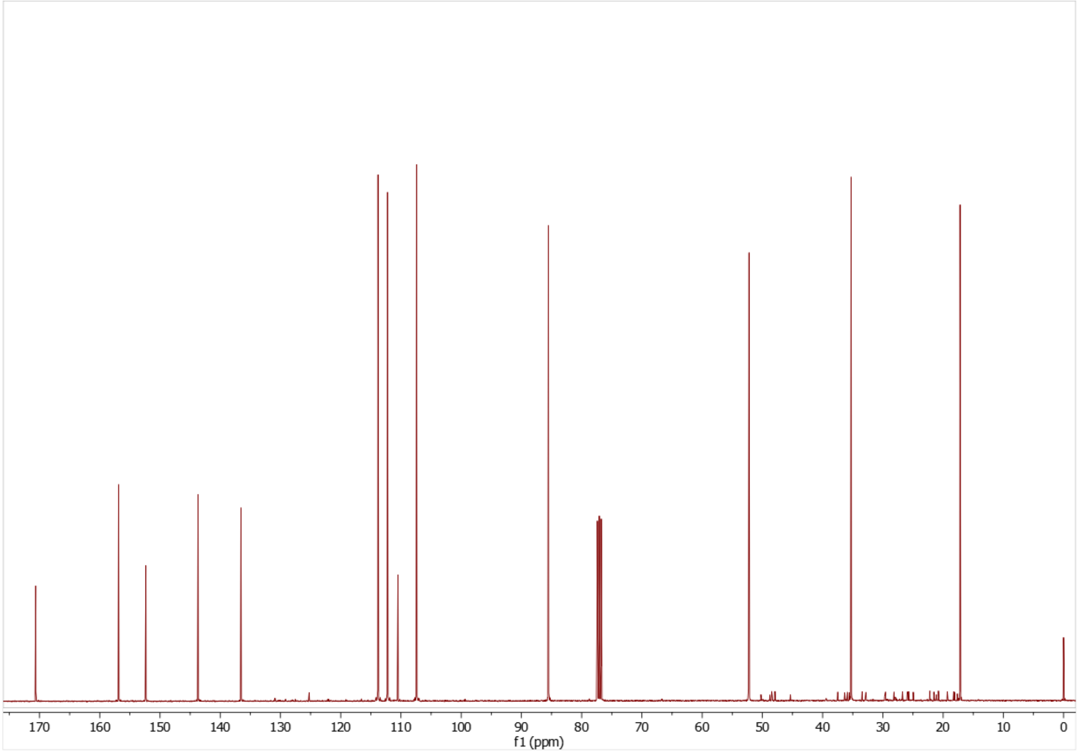

Figure S2. DEPT 135 NMR spectrum of fraction F2.5 (compound **1**).

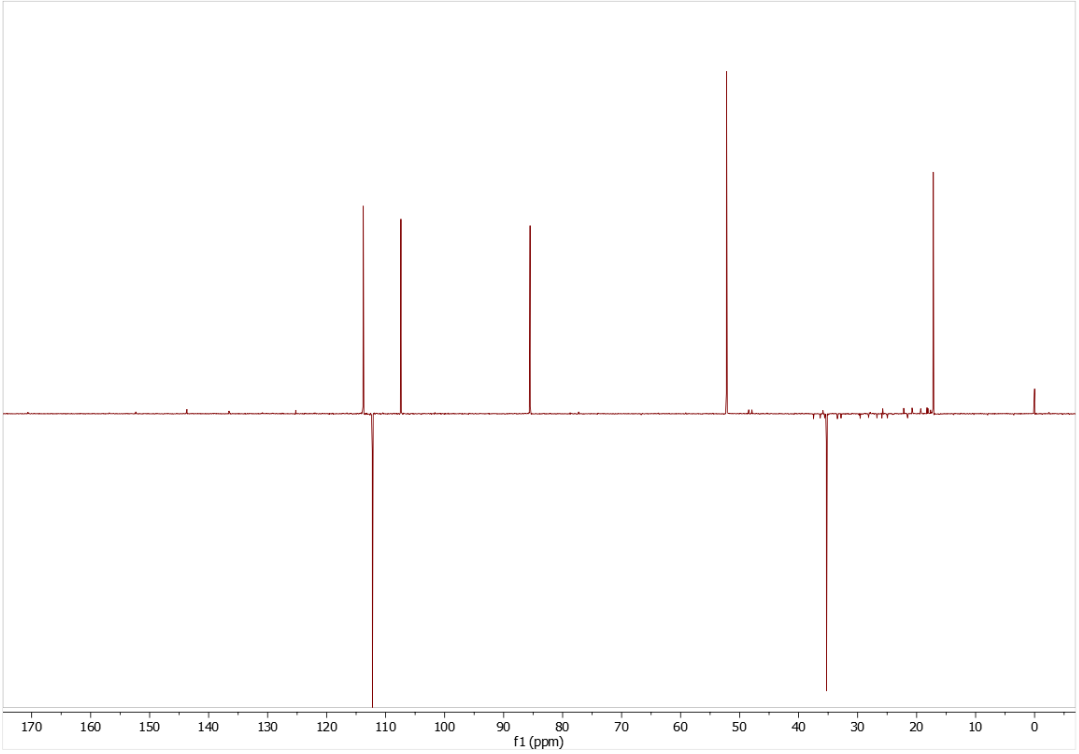

Figure S3. ^{1}H NMR spectrum of fraction F2.5 (compound **1**).

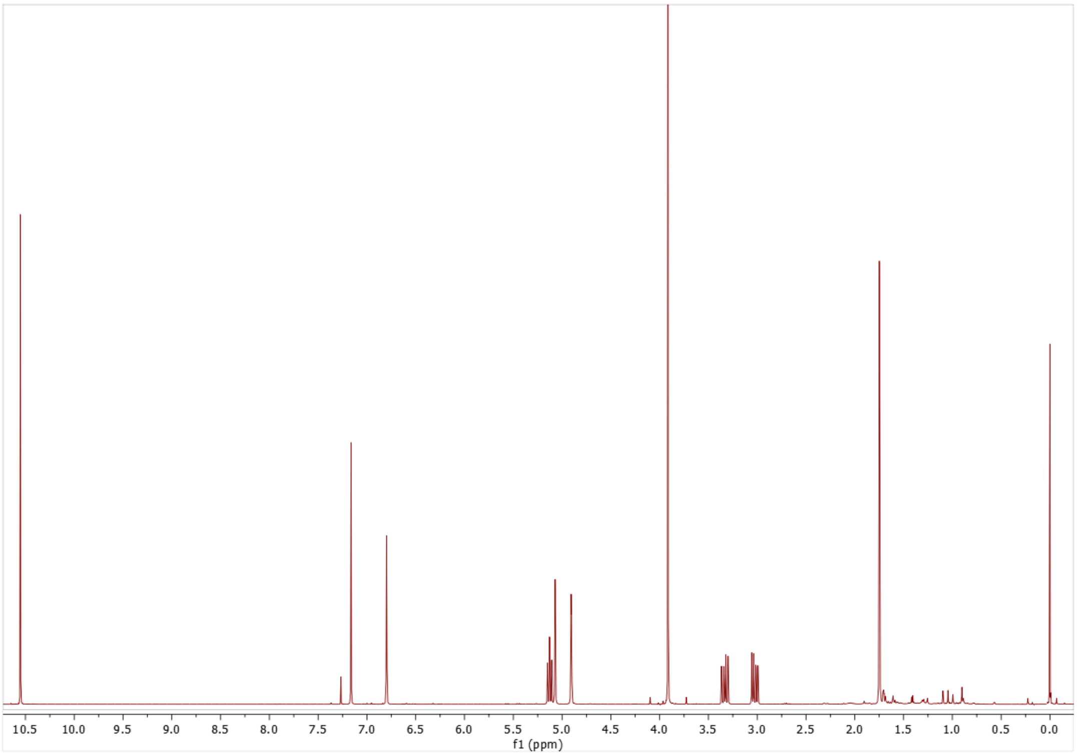

Figure S4. Structure of compound **1** with key HMBC and COSY correlations.

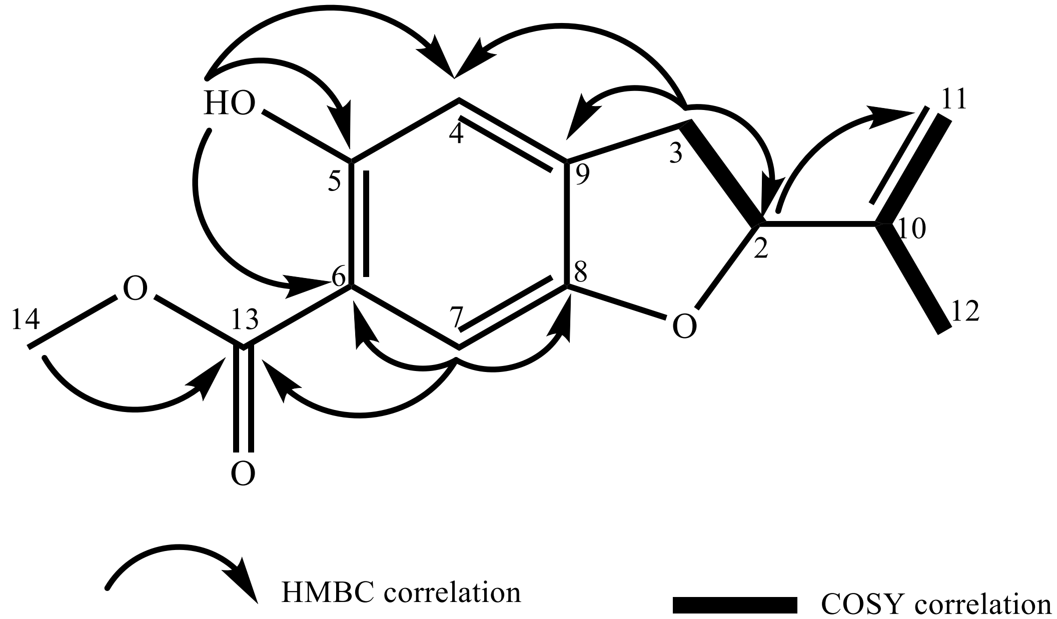

Figure S5. HSQC NMR spectrum of fraction F2.5 (compound **1**).

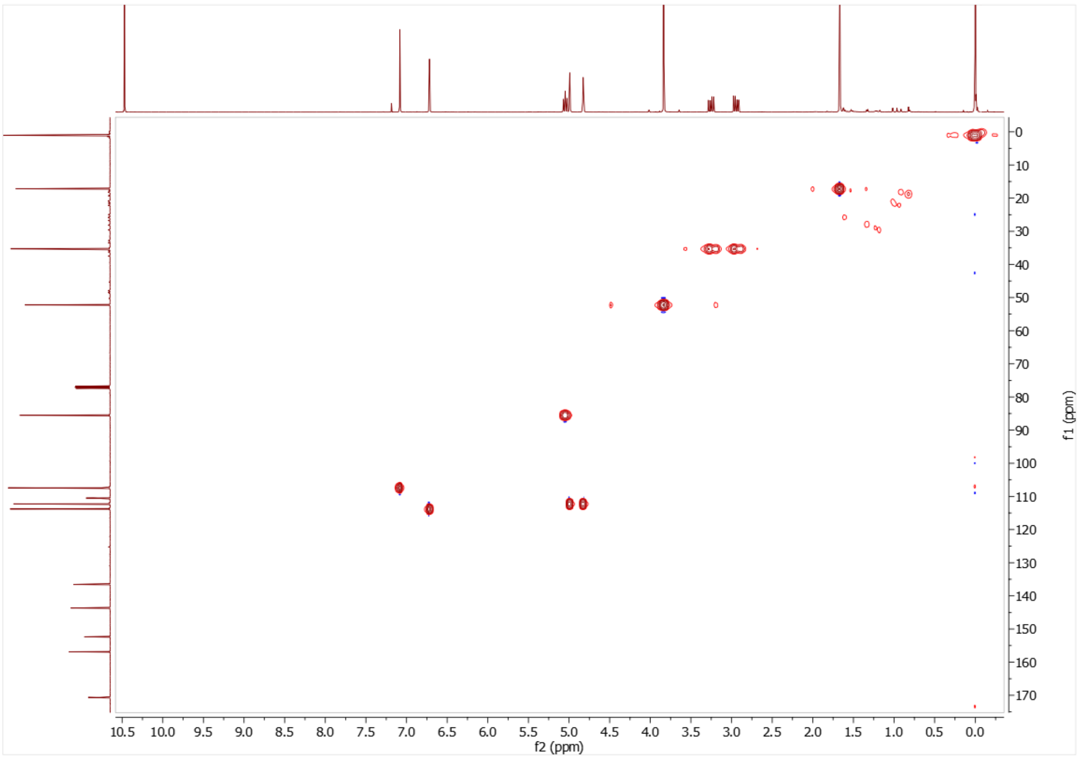

Figure S6. HMBC NMR spectrum of fraction F2.5 (compound **1**).

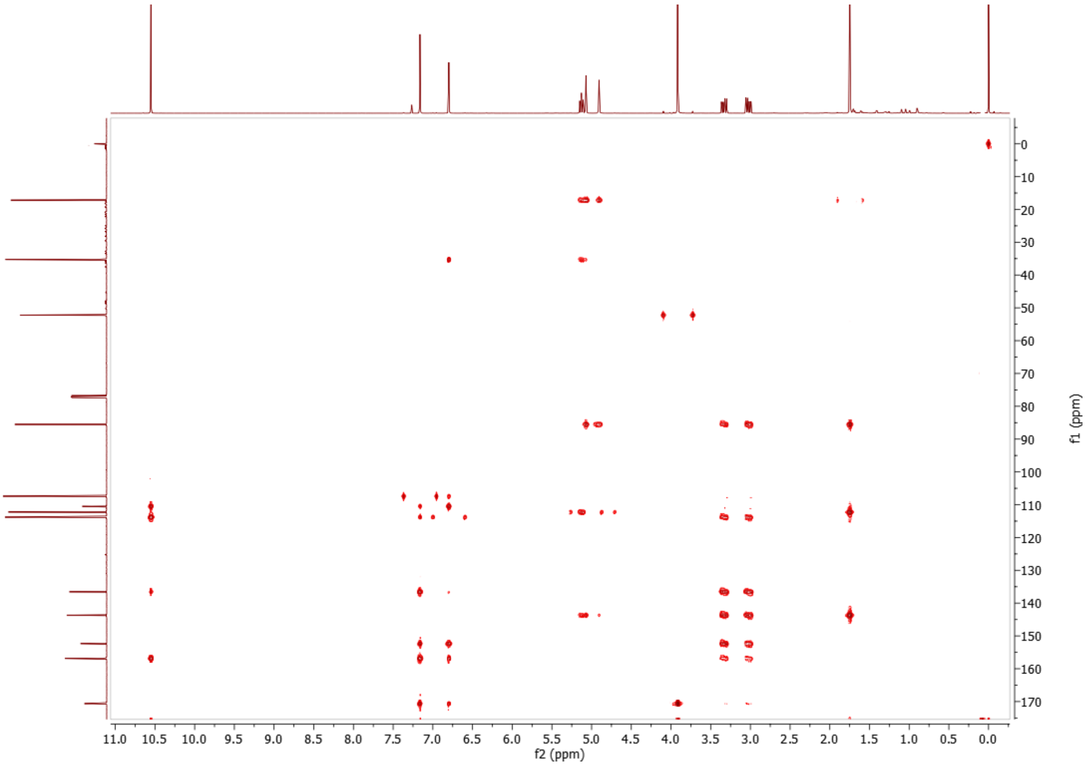

Figure S7. COSY NMR spectrum of fraction F2.5 (compound **1**).

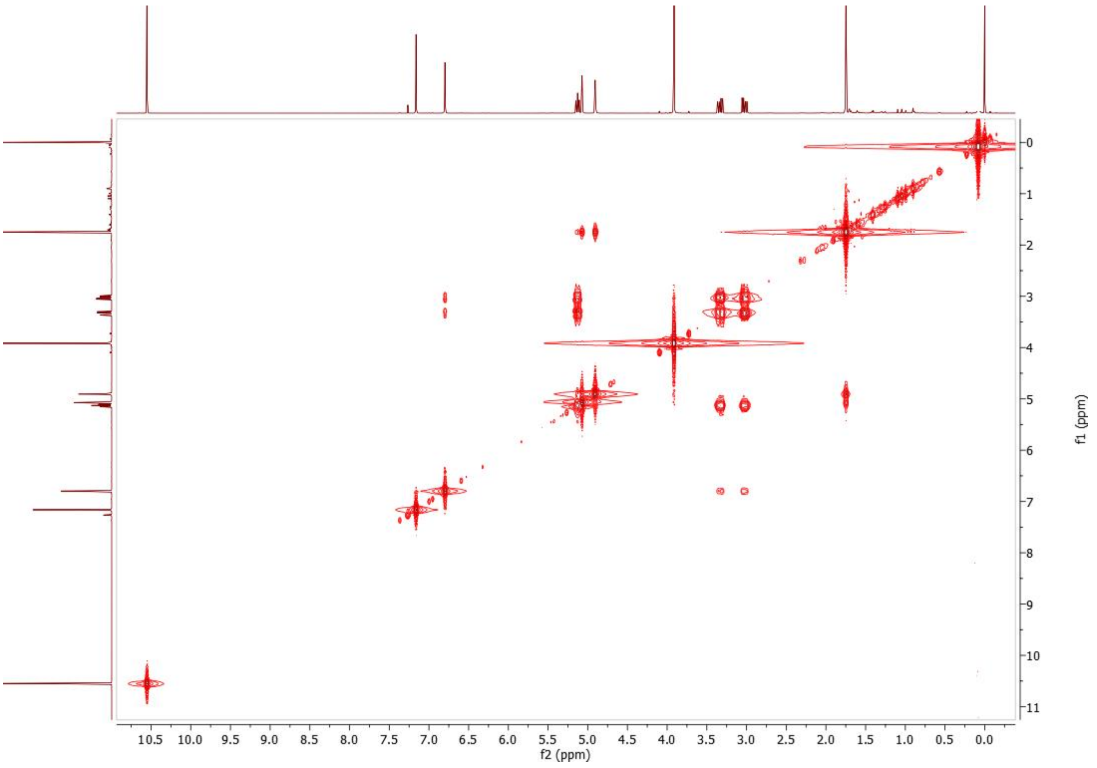

Figure S8. NOESY NMR spectrum of fraction F2.5 (compound **1**).

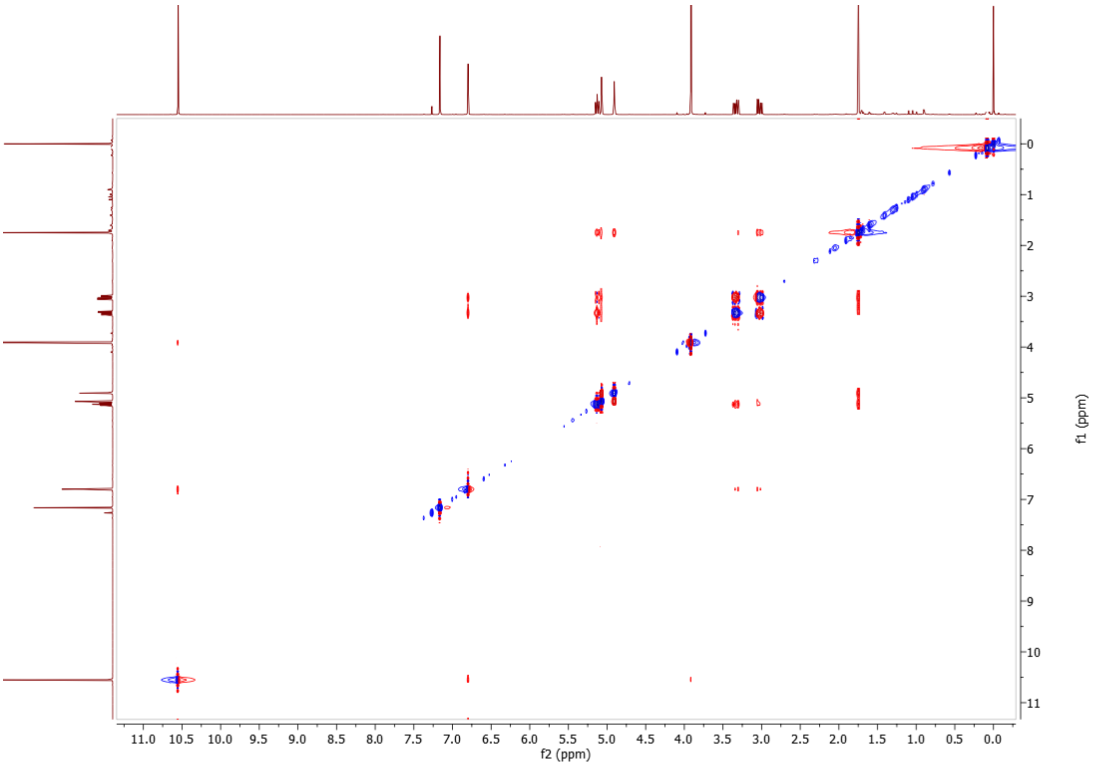

Figure S9. Structure of compound **1** with 2D NMR INADEQUATE correlations.

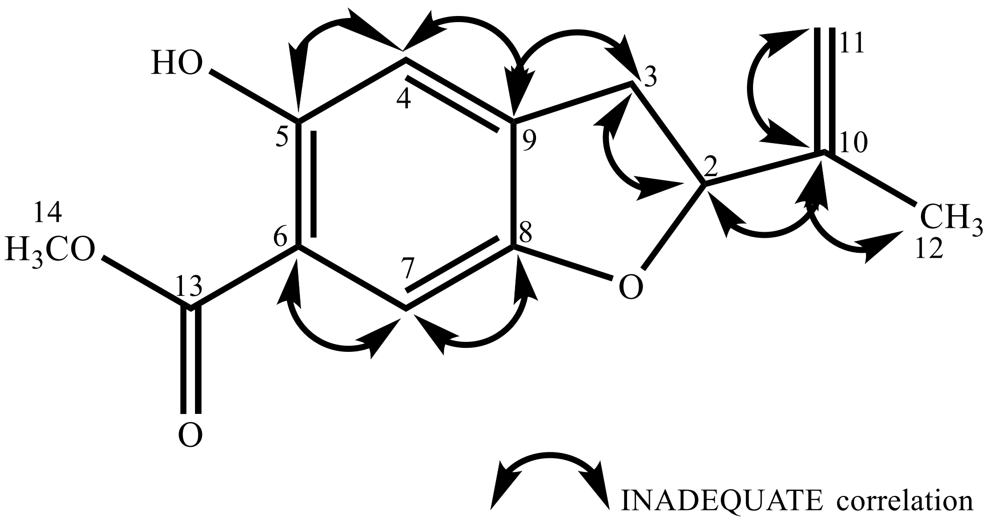

Figure S10. INADEQUATE NMR spectrum of fraction F2.5 (compound **1**).

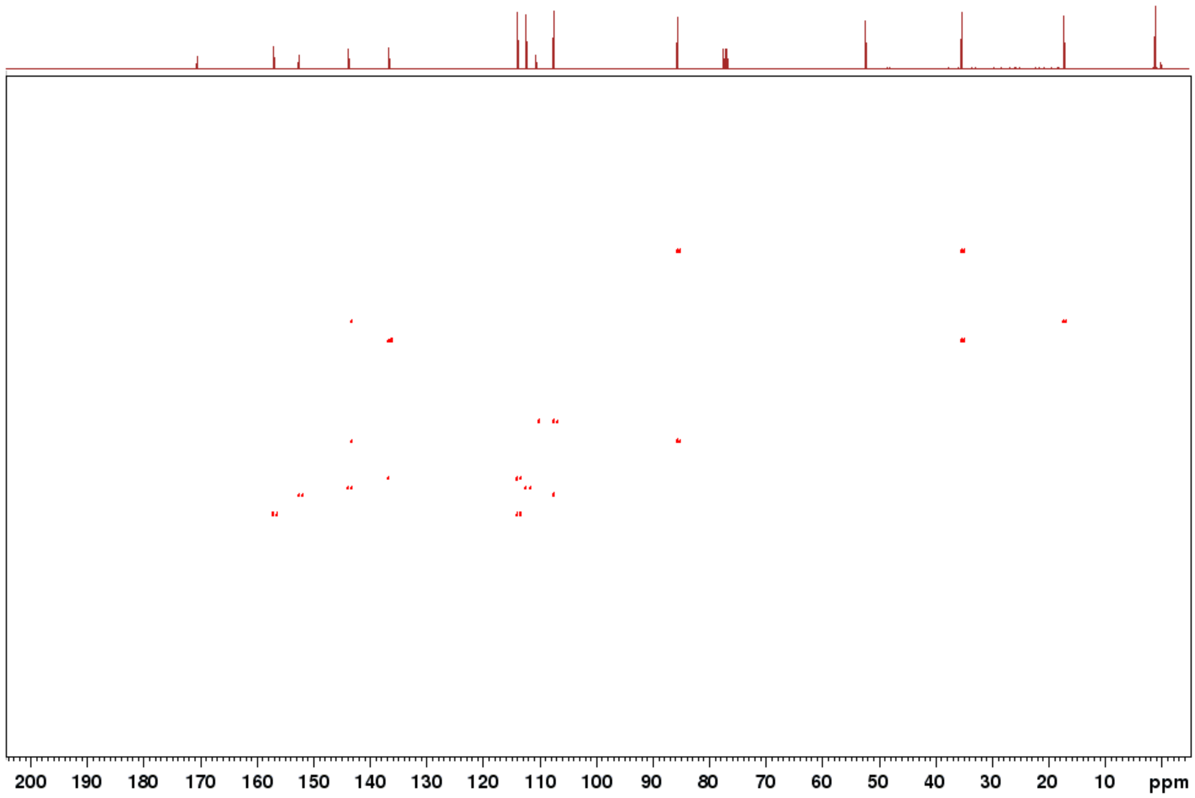

Figure S11. MS/MS spectrum of compound **1**.

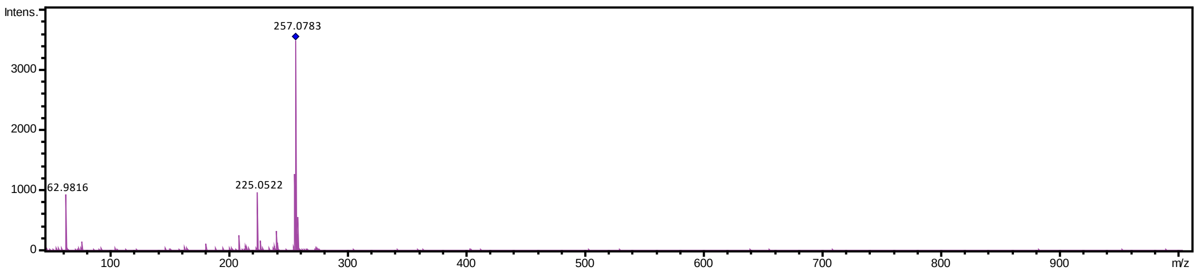

Figure S12. Structure of compound **2** with key HMBC and COSY correlations.

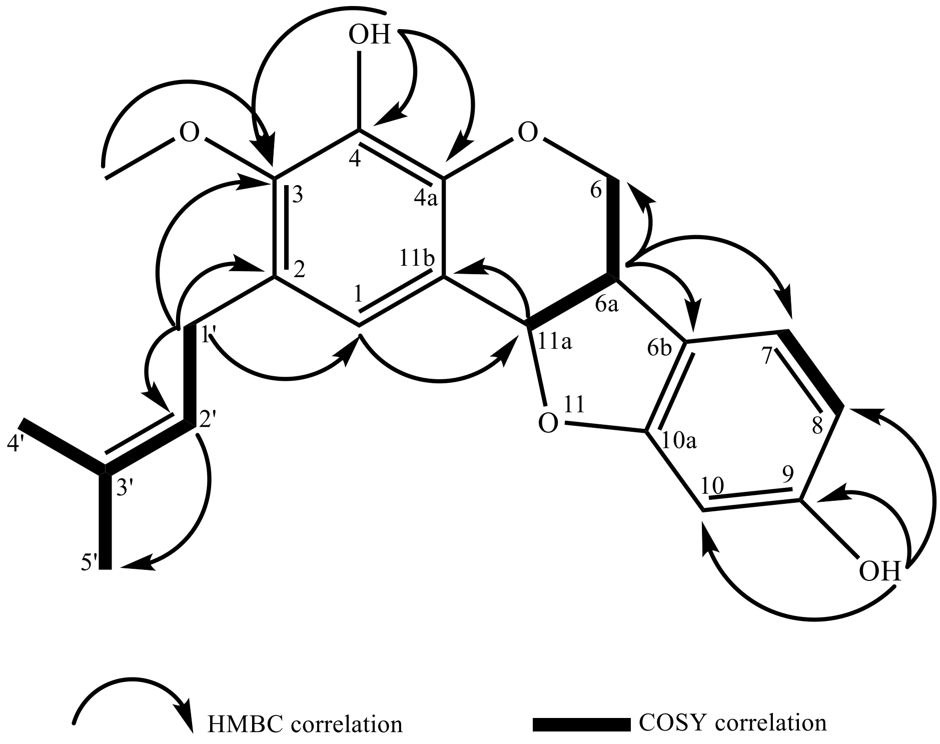

Figure S13. ^{1}H NMR spectrum of sub-fraction F3.B.3 (compound **2** / compound **3** = 3:1).

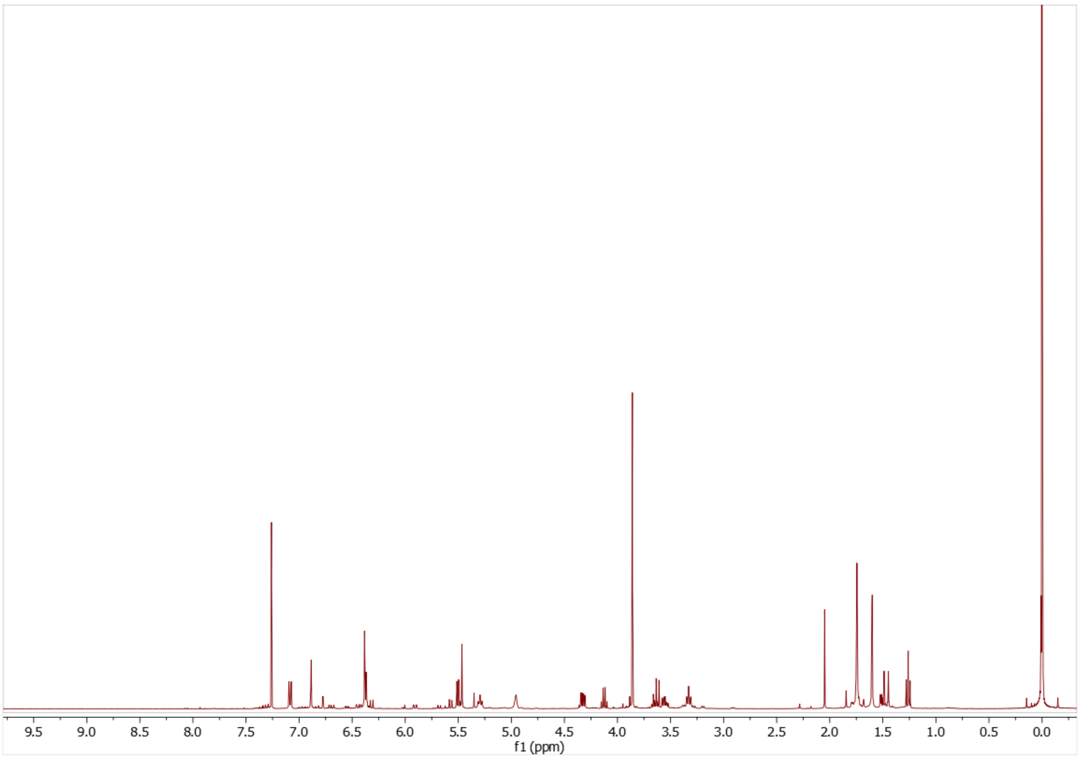

Figure S14. ^{13}C NMR spectrum of sub-fraction F3.B.3 (compound **2** / compound **3** = 3:1).

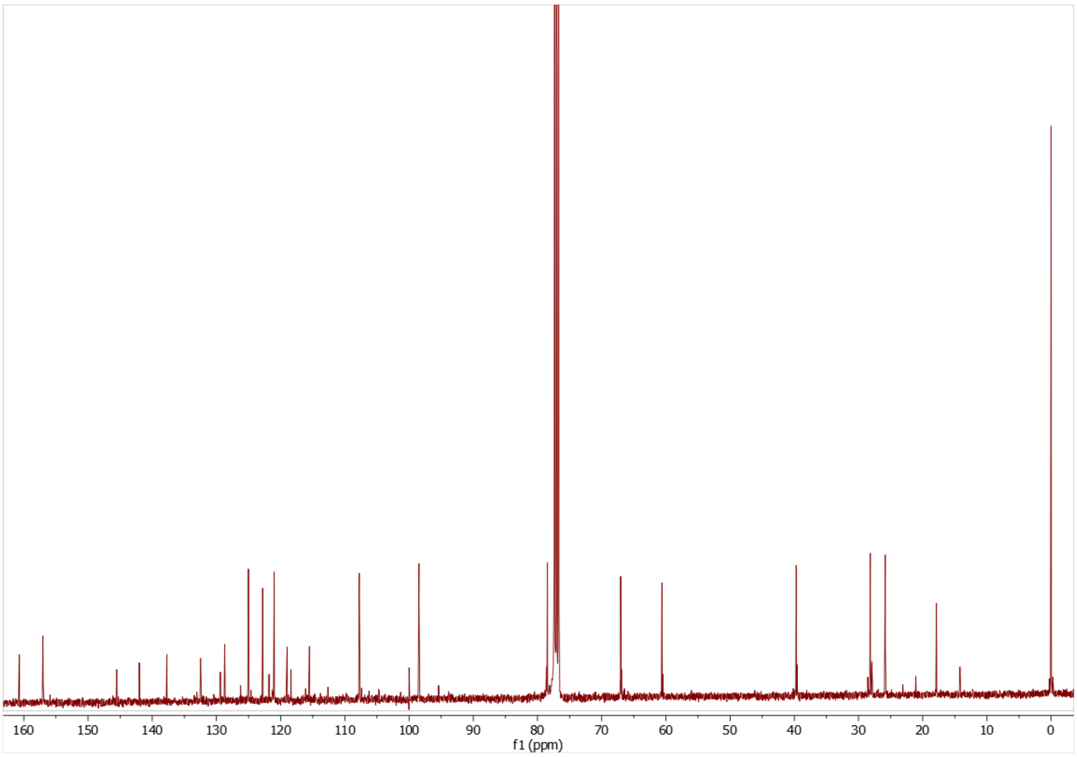

Figure S15. DEPT 135 NMR spectrum of sub-fraction F3.B.3 (compound **2** / compound **3** = 3:1).

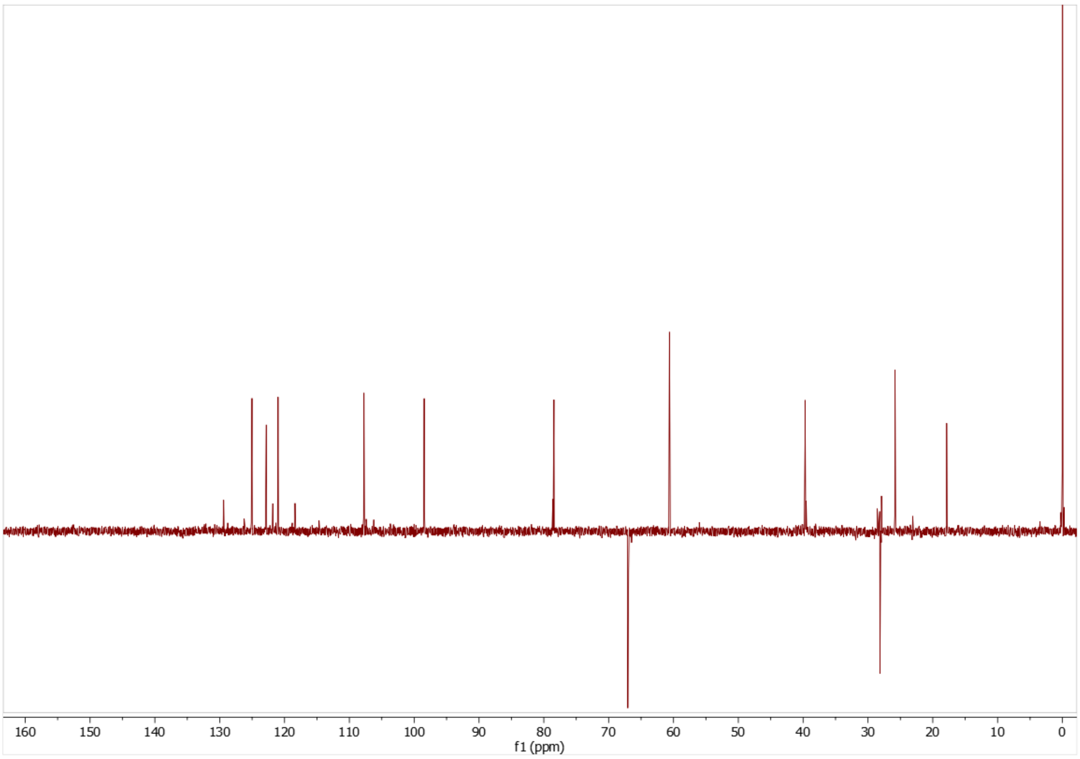

Figure S16. HSQC NMR spectrum of sub-fraction F3.B.3 (compound **2** / compound **3** = 3:1).

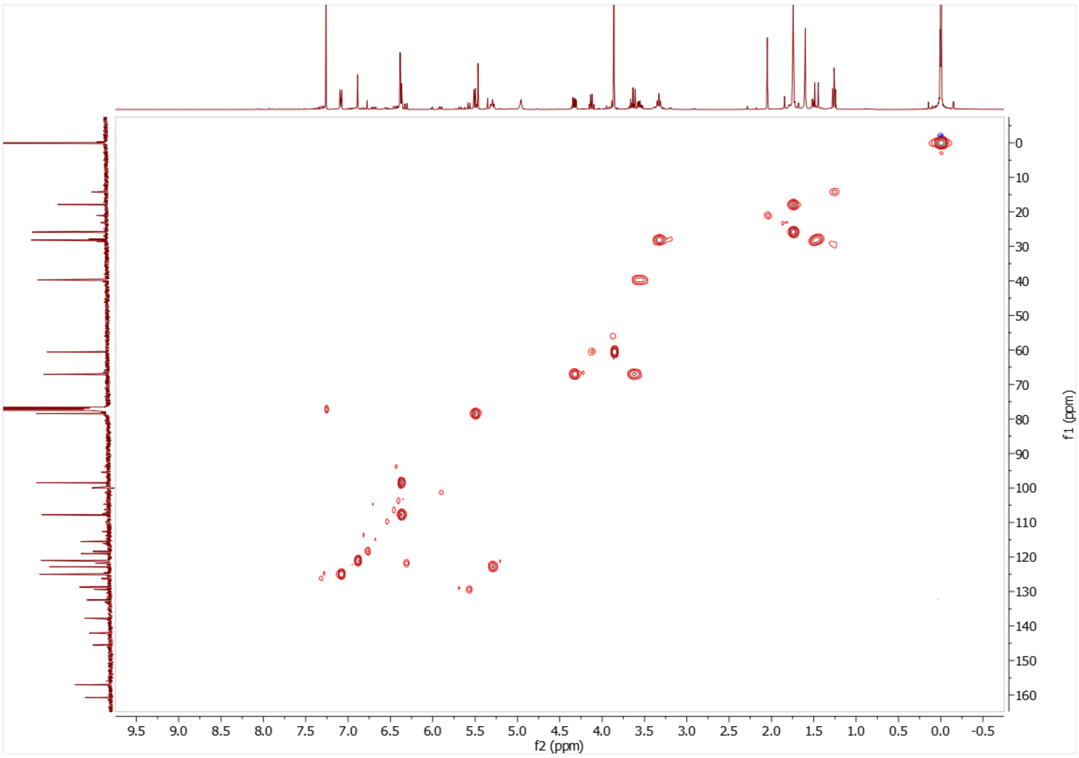

Figure S17. HMBC NMR spectrum of sub-fraction F3.B.3 (compound **2** / compound **3** = 3:1).

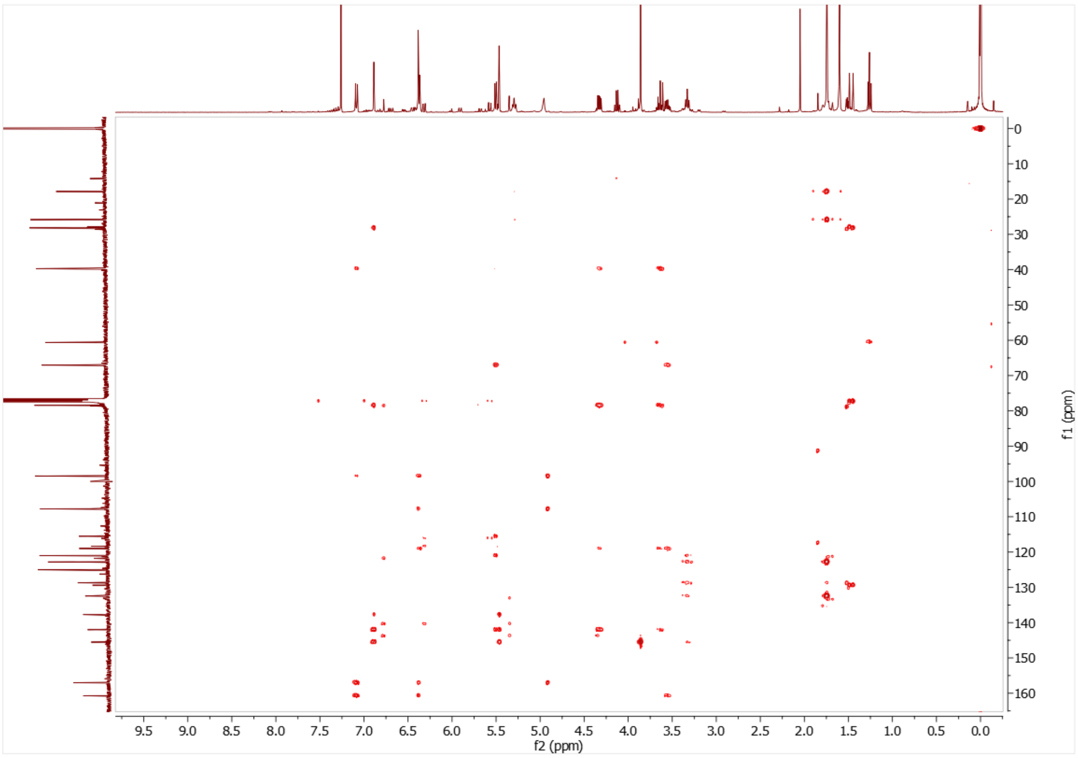

Figure S18. COSY NMR spectrum of sub-fraction F3.B.3 (compound **2** / compound **3** = 3:1).

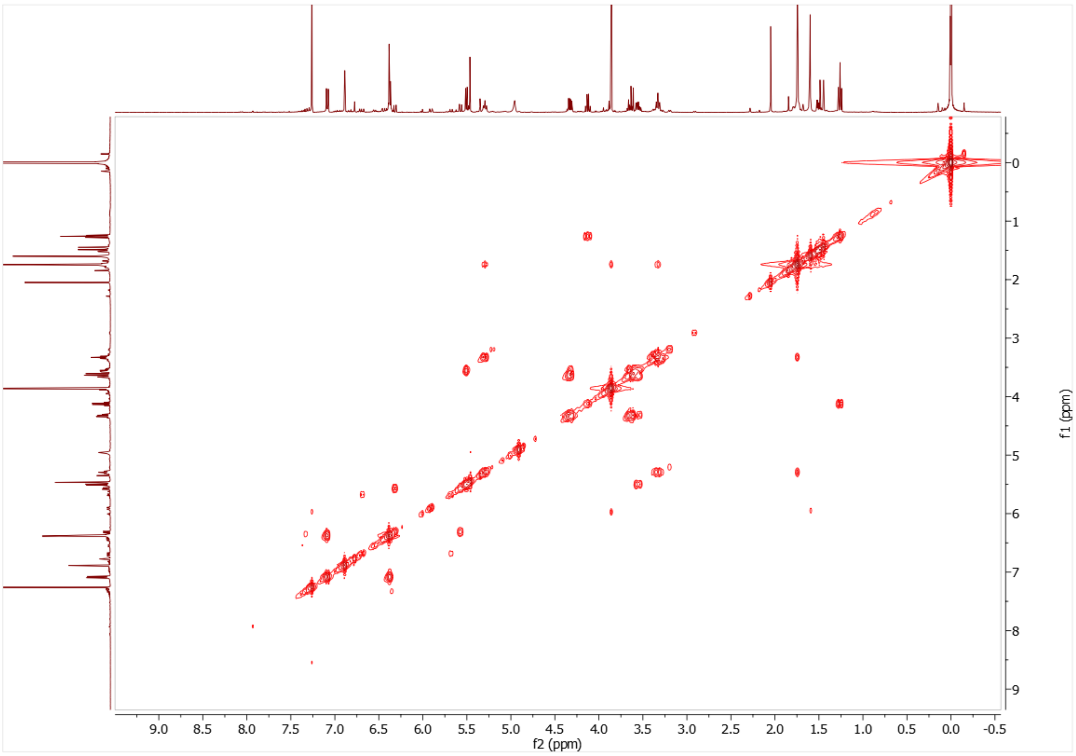

Figure S19. NOESY NMR spectrum of sub-fraction F3.B.3 (compound **2** / compound **3** = 3:1).

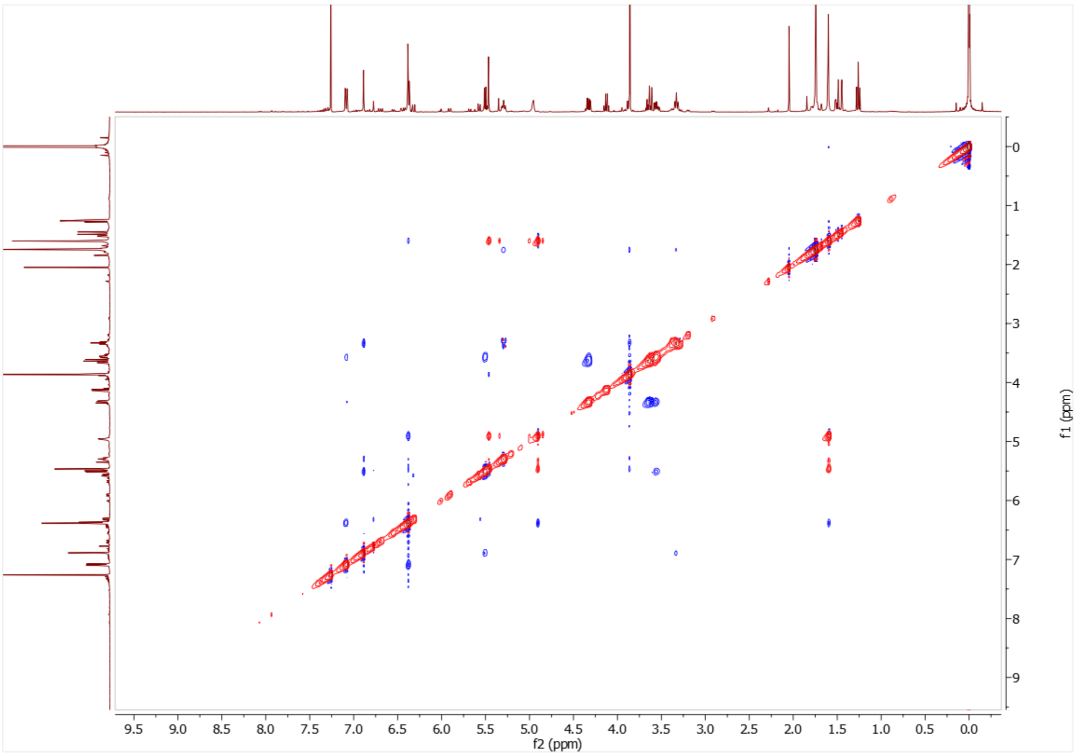

Figure S20. MS/MS spectrum of compound **2**.

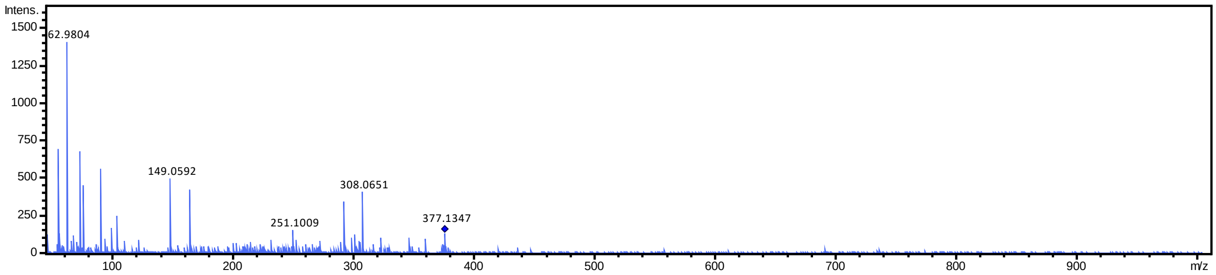

Figure S21. Structure of compound **3** with key HMBC and COSY correlations.

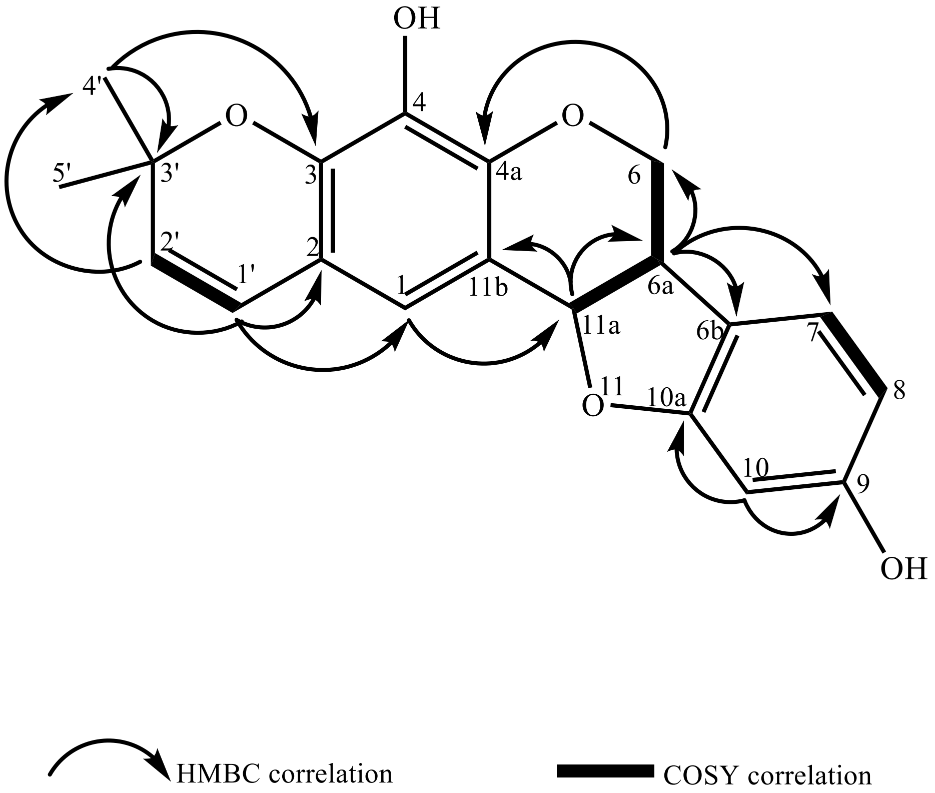

Figure S22. ^{1}H NMR spectrum of sub-fraction F3.B.7 (compound **2** / compound **3** = 1:4).

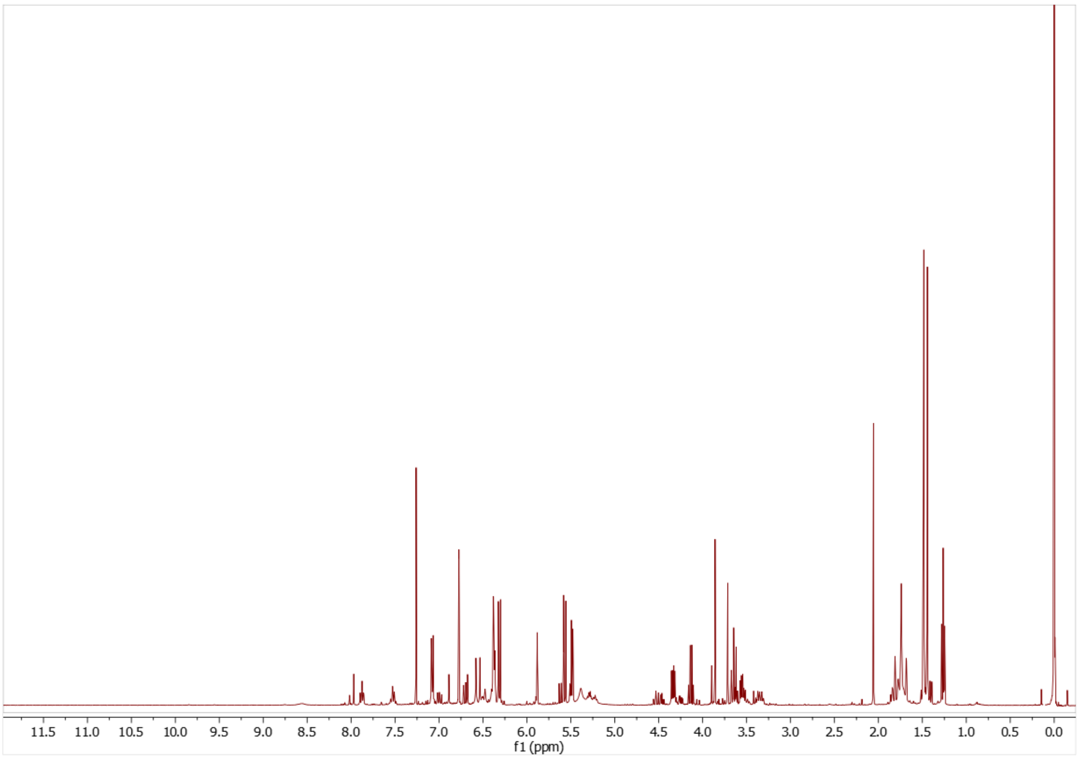

Figure S23. ^{13}C NMR spectrum of sub-fraction F3.B.7 (compound **2** / compound **3** = 1:4).

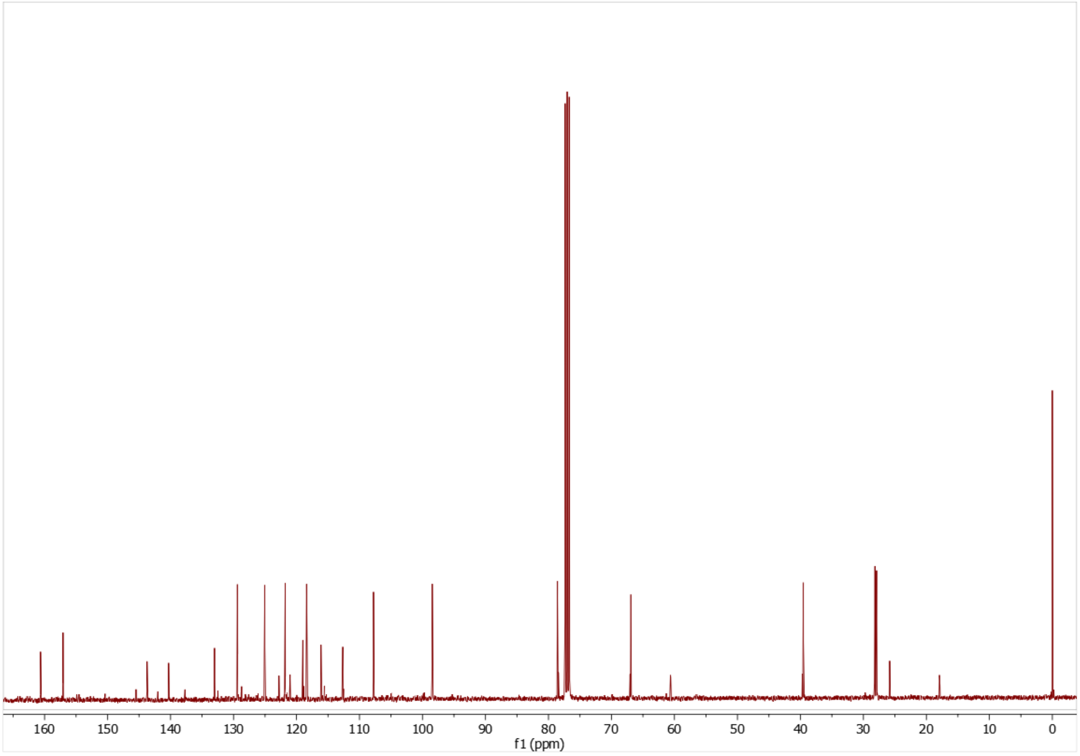

Figure S24. DEPT 135 NMR spectrum of sub-fraction F3.B.7 (compound **2** / compound **3** = 1:4).

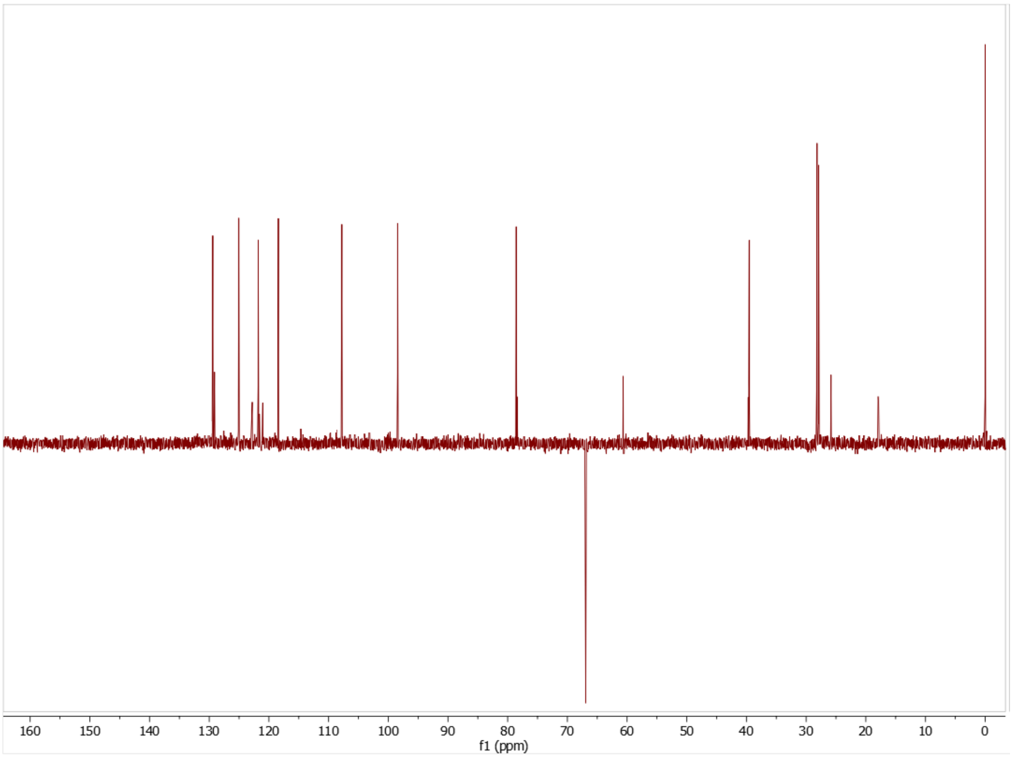

Figure S25. HSQC NMR spectrum of sub-fraction F3.B.7 (compound **2** / compound **3** = 1:4).

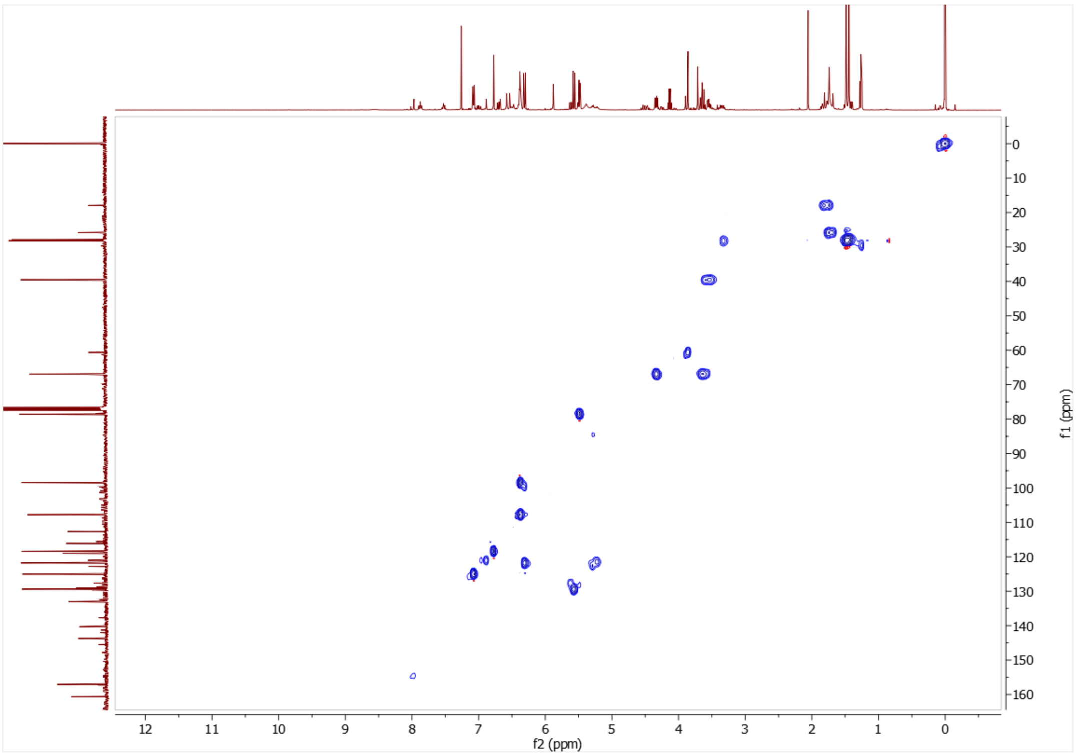

Figure S26. HMBC NMR spectrum of sub-fraction F3.B.7 (compound **2** / compound **3** = 1:4).

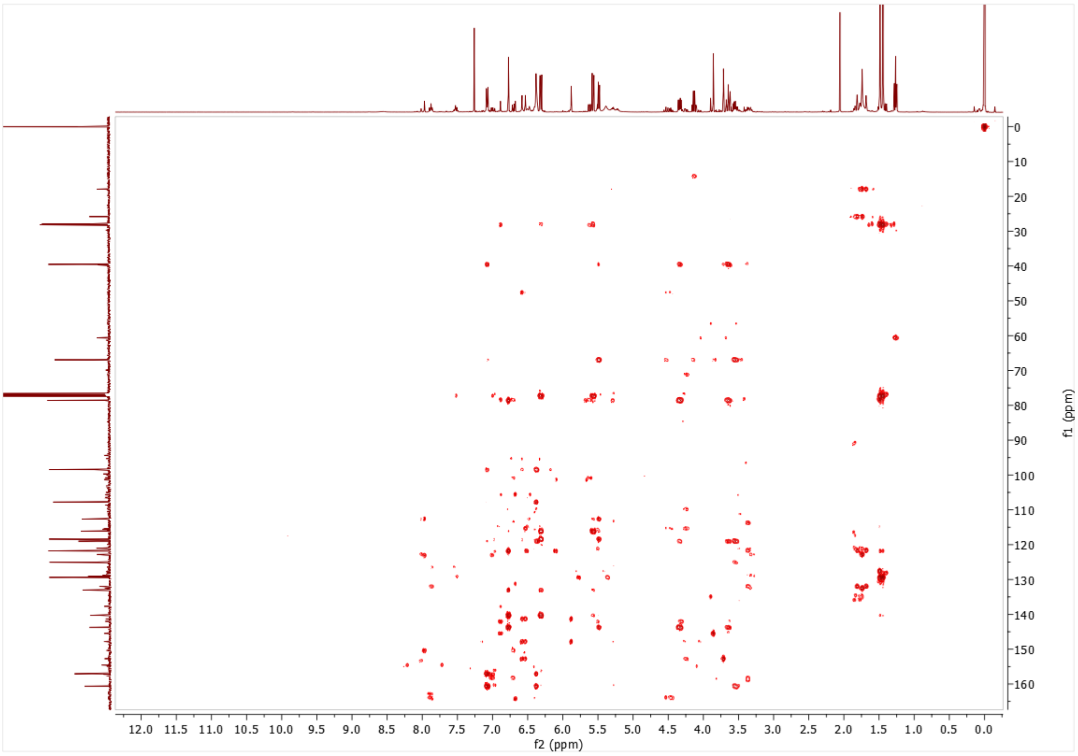

Figure S27. COSY NMR spectrum of sub-fraction F3.B.7 (compound **2** / compound **3** = 1:4).

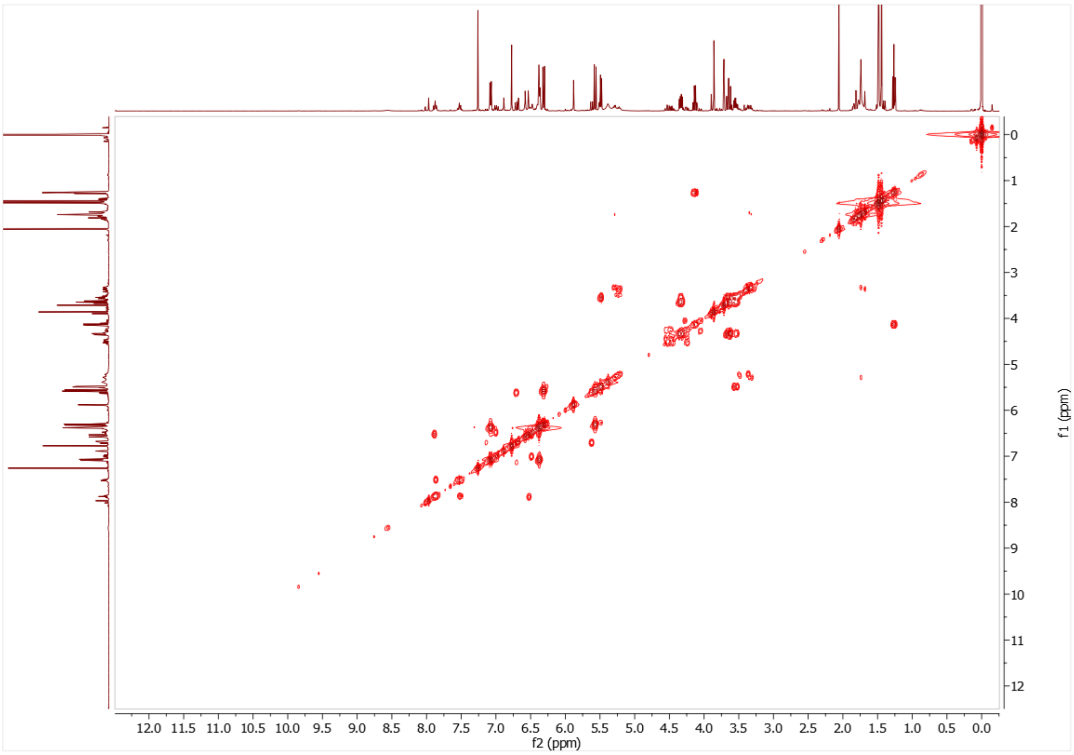

Figure S28. NOESY NMR spectrum of sub-fraction F3.B.7 (compound **2** / compound **3** = 1:4).

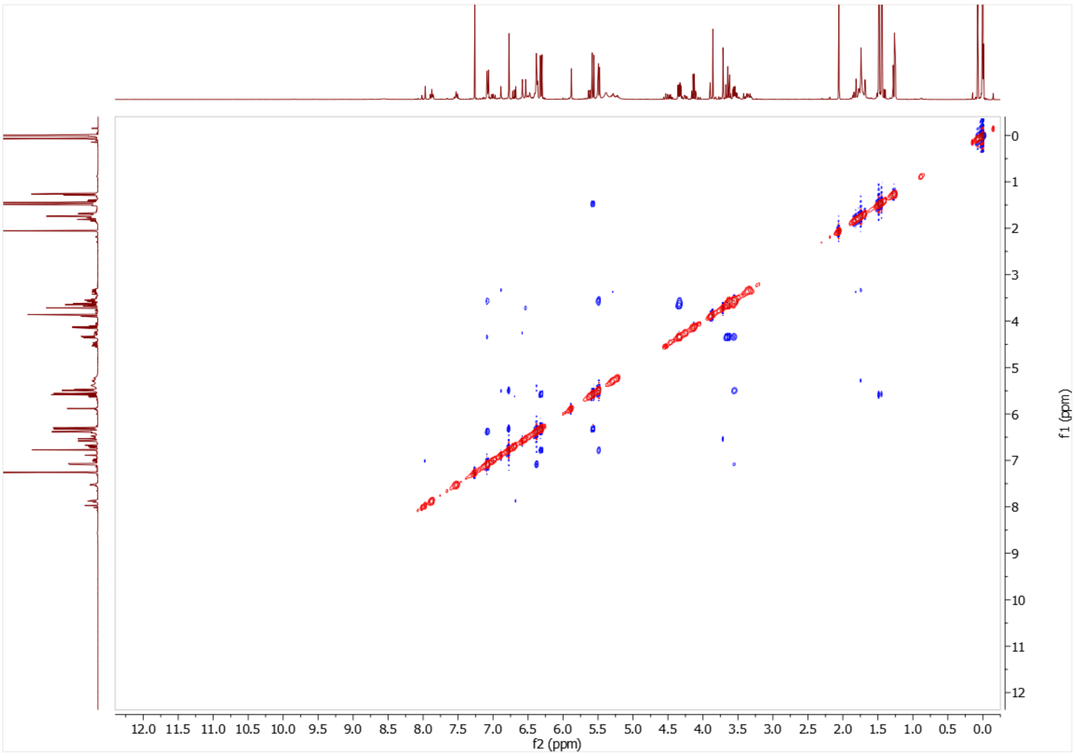

Figure S29. MS/MS spectrum of compound **3**.

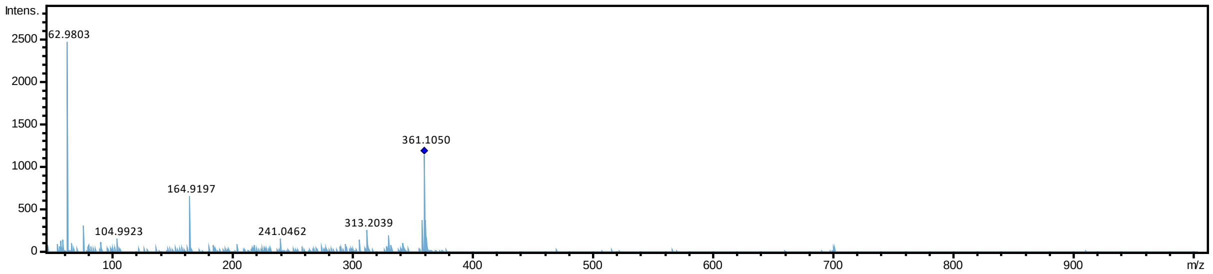

Figure S30. Possible pathway cleavages of compounds **2** and **3**.

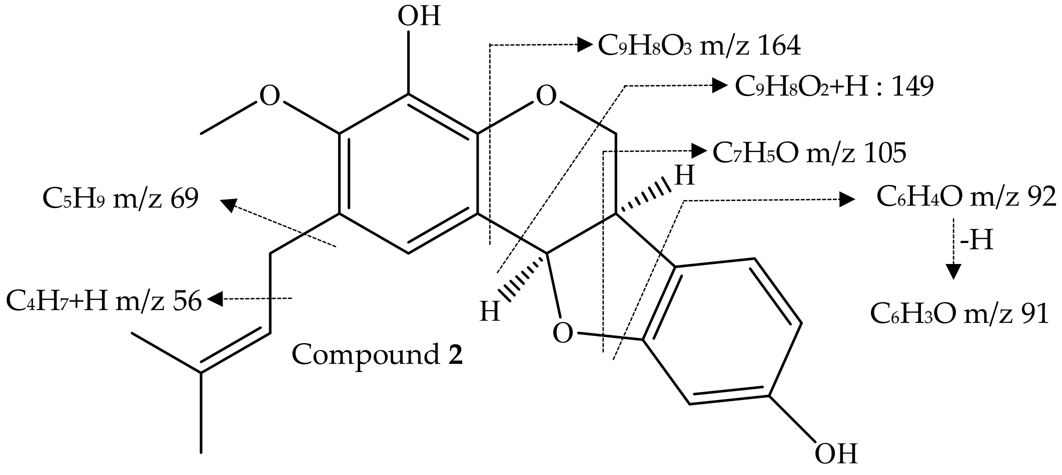

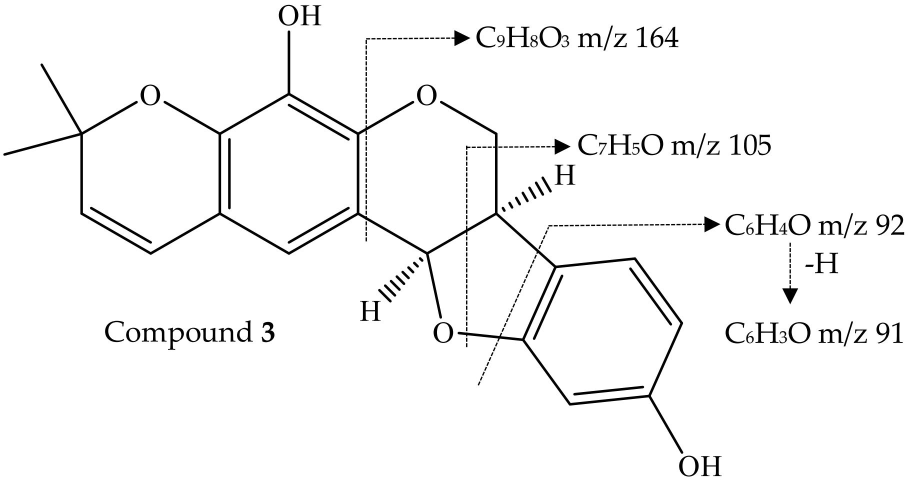

Supplement: Supplementary file 1 [file molecules-25-03467-s001.pdf]
